# Types of the cerebral arterial circle (circle of Willis) in a Sri Lankan Population

**DOI:** 10.1186/1471-2377-11-5

**Published:** 2011-01-17

**Authors:** K Ranil D De Silva, Rukmal Silva, Dhammika Amaratunga, WSL Gunasekera, Rohan W Jayesekera

**Affiliations:** 1 Department of Anatomy, Faculty of Medical Sciences, University of Sri Jayewardenepura, Nugegoda, Sri Lanka; 2 Department of Nonclinical Statistics, Johnson & Johnson Pharmaceutical Research & Development, Raritan, NJ, USA; 3 National Hospital of Sri Lanka, Colombo, Sri Lanka; 4 Department of Anatomy, Faculty of Medicine, University of Colombo, Colombo, Sri Lanka

## Abstract

**Background:**

The variations of the circle of Willis (CW) are clinically important as patients with effective collateral circulations have a lower risk of transient ischemic attack and stroke than those with ineffective collaterals. The aim of the present cadaveric study was to investigate the anatomical variations of the CW and to compare the frequency of prevalence of the different variations with previous autopsy studies as variations in the anatomy of the CW as a whole have not been studied in the Indian subcontinent.

**Methods:**

The external diameter of all the arteries forming the CW in 225 normal Sri Lankan adult cadaver brains was measured using a calibrated grid to determine the prevalence in the variation in CW. Chisquared tests and a correspondence analysis were performed to compare the relative frequencies of prevalence of anatomical variations in the CW across 6 studies of diverse ethnic populations.

**Results:**

We report 15 types of variations of CW out of 22 types previously described and one additional type: hypoplastic precommunicating part of the anterior cerebral arteries (A1) and contralateral posterior communicating arteries (PcoA) 5(2%). Statistically significant differences (p < 0.0001) were found between most of the studies except for the Moroccan study. An especially notable difference was observed in the following 4 configurations: 1) hypoplastic precommunicating part of the posterior cerebral arteries (P1), and contralateral A1, 2) hypoplastic PcoA and contralateral P1, 3) hypoplastic PcoA, anterior communicating artery (AcoA) and contralateral P1, 4) bilateral hypoplastic P1s and AcoA in a Caucasian dominant study by Fisher versus the rest of the studies.

**Conclusion:**

The present study reveals that there are significant variations in the CW among intra and inter ethnic groups (Caucasian, African and Asian: Iran and Sri Lanka dominant populations), and warrants further studies keeping the methods of measurements, data assessment, and the definitions of hypoplasia the same.

## Background

Based on anatomical [1-4] and radiological studies [5-8], it has been shown that more than 50% of healthy control subjects have anatomical variations in the circle of Willis (CW). Comparisons based on radiological studies [5-8] in living patients and anatomical autopsy studies [1-4] are not possible as in-vivo data from angiography record luminal diameters of vessels distended by normal arterial blood pressure, whereas the cadaveric studies report on external diameters of collapsed vessels with zero luminal pressures. The variations of the CW are clinically important as the CW plays an important role in cerebral hemodynamic as a collateral anastomotic network and patients with effective collateral circulations have a lower risk of transient ischemic attack and stroke than those with ineffective collaterals [9,10]. Fetal configuration [where the diameter of the ipsilateral pre-communicating (P1) segment of the posterior cerebral artery (PCA) is less than the diameter of PcoA, so that the blood supply to the occipital lobe is mainly via the internal carotid artery (ICA)] were found in autopsy brains with infarcts than in brains without [11,12]. Studies have shown that there also exists a correlation between cerebral aneurysms and certain variations of the CW [13-15].

Several studies [2,16-19] have reported a range of variations in the anatomy of the CW as a whole, but it is not clear whether the frequency of occurrence of the different variations of the CW are similar in the studies done in the Indian subcontinent as compared to studies done in other ethnic or racial populations. The range of variations in the anatomy of the CW has not been previously studied in Sri Lanka and the aim of this cadaveric study was to investigate the anatomical variations of the CW in subjects who have died of causes unrelated to the brain and to compare the frequency of prevalence of the different variations with previous autopsy studies.

## Methods

225 postmortem brains (184 male and 41 female) were obtained following ethical approval from the Ethics Committee of the Faculty of Medicine, Colombo from medicolegal autopsies in individuals aged between 18 and 73 years who have died of causes unrelated to the brain. The brains were removed from the cranial cavity and fixed in 10% formaldehyde for a minimum period of two weeks. The arteries comprising the CW together with the basilar artery and its minute branches arising from the main vessels were then carefully removed from the base of the brain. Blood was carefully washed out from the CW with isotonic saline. Line diagrams of all 225 circles were obtained, including photographic records in some cases.

Segments were taken from the following corresponding regions: right and left internal carotid arteries (ICA) close to their distal ends, precommunicating and postcommunicating part of the anterior cerebral arteries (A1), (A2) and the posterior cerebral arteries (P1), (P2) close to their origin, right and left posterior communicating arteries (PcoA) at their middle point and anterior communicating artery (AcoA) (with its variations if present) at its middle point. Transverse sections were cut (at 40 um) from each of the segments obtained as stated above in a plane that was perpendicular to the vessel (microtome model Shandon M1R, UK); a random 'section' was then obtained from the water bath and three measurements of the external diameter were performed on each section by the first Author under a stereomicroscope equipped with a micrometer-calibrator (Leica, WILD M3B, Stereomicroscope). The calculated average was then recorded as the value for each artery. Arteries where the external diameter was less than 1 mm, were documented as <1 mm. The equipment was standardised according to the manufacturer's specifications.

**Figure 1:**
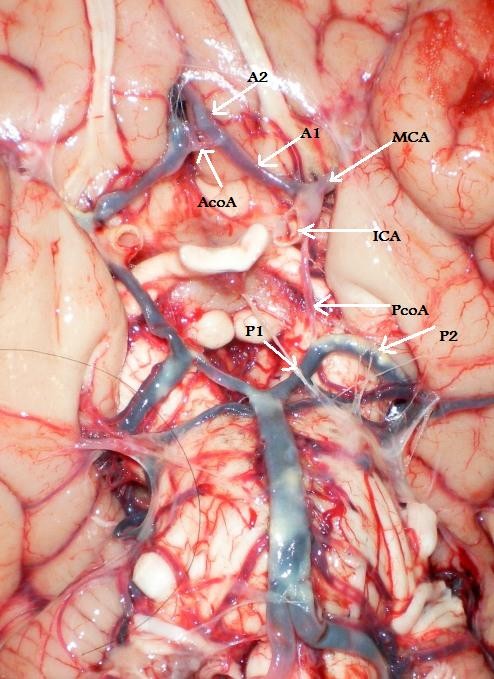
**(Type 1) - "Typical CW"**.

In the present study, the CW was defined as "typical CW" (Figure 1) only if:

1. All the component vessels (*i.e. *ICA, A1, AcoA, PcoA and P1 arteries) were present.

2. Origin of the arteries forming the CW was from its normal source with no excess vessels.

3. The external diameter of a component artery was not less than one millimeter.

**Figure 2:**
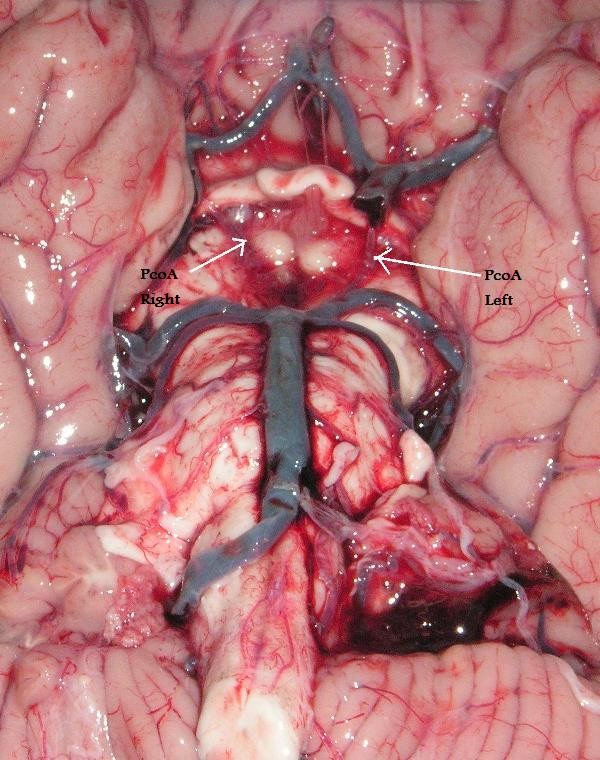
**(Type 6) - Bilateral hypoplastic PcoA**.

External diameter less than 1 mm. in any artery was considered to be "hypoplastic" (string-like appearance) (Figure 2), in order to be consistent with many previous anatomical studies [12,17,19,20]. A vessel was recorded as "absent" only when it was not visualized following examination under the dissecting microscope.

There are many anatomical variations of the CW, their classification into a few clearly arranged groups is hardly possible, we classified variations of CW using 22 Types as defined by Lazorthes *et al*.,1979, [18] and Eftekhar *et al.*, 1985 [19]. To the best of our knowledge only six studies [2,3,16-19] and the present study have investigated variations of CW as a whole and have classified all vessels with diameter under 1 mm as hypoplastic, but the selection of cases, nomenclature and the methodology adapted to measure the external diameter of the vessels were not identical.

### Statistical methods

In order to study the relationship of the anatomical variations of the CW between the studies in Caucasian dominant (USA [2,17] and France [18]), African (Morocco) [16] and Asian (Iran, [19] and present study from Sri Lanka) we performed the following analyses. First, a global chisquared test was used to compare across all 6 studies, and then a series of pairwise chisquared tests was used to test for differences between each pair of studies. Since there are several small counts in the data, it is possible that the chi-squared distributional approximation would be inadequate; therefore exact tests were used (after removing any zero marginals) with one million random permutations for each test [21]. A Bonferroni adjustment was applied to address the multiple testing issue.

To supplement these pairwise comparisons, a more global (i.e., non-pairwise) analysis was also performed to better assess study similarities and dissimilarities and also, for those studies that differ, to determine which configurations are primarily responsible for the difference. This was done using correspondence analysis. Correspondence analysis (proposed by Benzecri (1969) [22] and subsequently reviewed by Greenacre (2007) [23] and Nishisato (1980) [24]) is a statistical method for studying associations between the levels of the rows and columns of a two-way contingency table by performing a singular value decomposition (essentially a form of principal component analysis) of the table. The result of a correspondence analysis is a two-dimensional graphical representation of the association between rows and columns of the table. The plot contains a point for each row of the table and a point for each column of the table. Rows with similar patterns of counts produce row points that are close together and columns with similar patterns of counts produce column points that are close together. In addition, if certain row points and certain column points separate in a particular direction, then the levels corresponding to those points are likely to be associated.

## Results

We report 15 types of variations of CW out of 22 types previously described by Lazorthes *et al*.,1979, [18] and Eftekhar *et al.*, 1985 [19] and one additional type: hypoplastic A1 and contralateral PcoA 5(2.2%), categorized under "others" in Table 1. Variations in the CW in the present study are shown in table 1. The most common variations are as follows:

**Table 1:** Comparison of the variations of the CW

	Author	**Riggs and Rupp 1963,**[2]	**El Khamlichi *et al*., 1985, **[16]	**Fisher 1965,**[17]	**Lazorthes *et al*., 1979,**[18]	**Eftekhar *et al.*, 2006,**[19]	Present study
	Country	USA (1)	Morocco	USA (2)	France	Iran	Sri Lanka
	Total brains	994	100	414	200	102^b^	225
Types	Configuration						
1	Typical	192(19)	18(18)	20(5)	29(14.5)	29(28)	32(14)
2	all segments hypoplastic	54(5)	0(0)	0(0)	10(5)	0(0)	0(0)
3	hypoplastic AcoA	91(9)	11(11)	6(1)	9(4.5)	0(0)	32(14)
4	Unilateral hypoplastic PcoA	88(9)	14(14)	24(6)	28(14)	20(20)	26(11.5)
5	Unilateral hypoplastic PcoA and AcoA	41(4)	6(6)	12(3)	10(5)	4(4)	15(7)
6	Bilateral hypoplastic PcoAs;	126(13)	24(24)	131(32)	44(22)	28(27)	52(23)
7	Bilateral hypoplastic PcoAs and hypoplastic AcoA	67(7)	10(10)	58(14)	34(17)	4(4)	37(16)
8	hypoplastic A1	38(5)	2(2)	0(0)	3(1.5)	0(0)	6 (3)
9	Unilateral hypoplastic P1	47(5)	3(3)	4(0.9)	5(2.5)	1(0.9)	2(0.8)
10	Bilateral hypoplastic P1s	33(3)	1(1)	16(4)	6(3)	0(0)	1(0.4)
11	hypoplastic P1 and contralateral A1	2(0.2)	0(0)	10(2)	0(0)	0(0)	0(0)
12	hypoplastic P1 and ipsilateral A1	20(2)	1(1)	1(0.2)	3(1.5)	1(0.9)	4 (2)
13	Bilateral hypoplastic P1s and A1	5(0.5)	0(0)	3(0.7)	1(0.5)	0(0)	1(0.4)
14	hypoplastic A1 and contralateral PcoA	7(0.7)	0(0)	1(0.2)	1(0.5)	0(0)	0(0)
15	Hypoplastic AcoA and P1	35(3.5)	4(4)	0(0)	4(2)	1(0.9)	6(3)
16	hypoplastic PcoA, ipsilateral A1 and AcoA	16(2)	3(3)	2(0.4)	2(1)	0(0)	0(0)
17	hypoplastic PcoA and contralateral P1	26(3)	0(0)	46(11)	3(1.5)	2(2)	1(0.4)
18	A1 and bilateral hypoplastic PcoAs	58(6)	0(0)	21(5.0)	6(3)	0(0)	4(2)
19	hypoplastic PcoA, AcoA and contralateral P1	17(2)	1(1)	28(7)	1(0.5)	1(0.9)	0(0)
20	hypoplastic P1, contralateral PcoA and ipsilateral A1	10(1)	1(1)	5(1)	0(0)	0(0)	0(0)
21	Bilateral hypoplastic P1s and AcoA	13(1)	0(0)	10(2)	1(0.5)	0(0)	1(0.4)
22	hypoplastic PcoA, ipsilateral A1 and contralateral P1	3(0.3)	0(0)	8(2)	0(0)	0(0)	0(0)
Others		5(0.5)	1(1)	8(2)	0(0)	1(0.9)	5(2)

(a) Column percentages are given in parentheses. (b) Eftekhar *et al. *2006[19] report the total in their study as 102 but findings are reported only for 92.

Type 1-"Typical CW": 32(14%); Type 3 - Hypoplastic AcoA: 32(14%); Type 4 - Unilateral hypoplastic PcoA: 26(11.5%); Type 5 - Unilateral hypoplastic PcoA and AcoA: 15(7%); Type 6 - Bilateral hypoplastic PcoAs, 52(23%); Type 7 - Bilateral hypoplastic PcoAs and hypoplastic AcoA: 37(16%).

### Statistical results

The global chi-squared test was highly significant (p < 0.0001), indicating differences among the studies. The pairwise p-values are given in Table 2 with comparisons significant at the 5% level denoted by an asterisk. Essentially all tests were significant except for Morocco [16] versus each of USA1, [2] France, [18] Iran, [19] and Sri Lanka.

**Table 2:** Results of the pairwise chi-squared tests (performed as exact tests)

Study	Morocco	USA2	France	Iran	Sri Lanka
USA1	0.6902	<0.0001*	0.0013*	0.0003*	<0.0001*
Morocco		<0.0001*	1.0000	0.0521	1.0000
USA2			<0.0001*	<0.0001*	<0.0001*
France				0.0054*	0.0115*
Iran					<0.0001*

The values shown are Bonferroni adjusted *p*-values. All values significant at the 5% level are shown with an asterisk.

**Figure 3:**
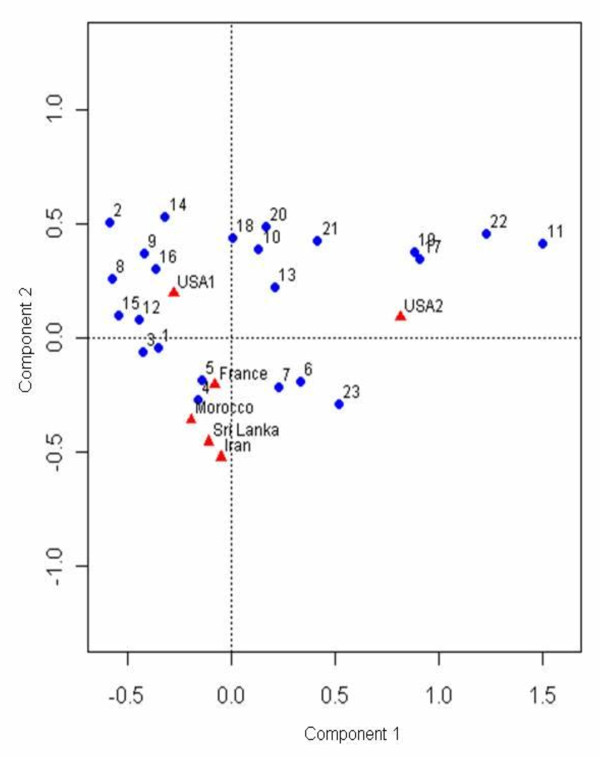
**Correspondence analysis plot of the data in Table 1**. The plot is a 2-dimensional representation of the 23-dimensional Countries data (the 6 filled circles) together with a 2-dimensional representation of the 6-dimensional Configuration Types data (the 23 unfilled circles). One notable observation is that USA2 separates from the rest of the Countries; since the separation is in the same direction as the deviation of Configuration Types numbered (in order from right to left) 11, 22, 17 and 19 from the center of the plot, these are the Configuration Types most associated with this separation; the proportions contributing to the separation can be read off from Table 1.

Thereafter, a correspondence analysis was used to study the degree of (dis)similarities among the studies; Figure 3; the 6 studies are represented by filled triangles and the 23 configurations are represented by filled circles.

## Discussion

Both the chi-squared analysis (Table 2) and the correspondence analysis (Figure 3) indicate that studies reported from Sri Lanka (i.e., the present study), Iran [19] and France [18] all have somewhat similar profiles (although the Sri Lanka vs France comparison is significant at the 5% level, the differences between the two are quite small as can be seen in Table 2). USA 2 [17] is clearly distinct from the rest driven by marked differences in configuration numbers 11, 17, 19 and 22 (note their respective locations in Figure 3). These 4 configurations, 11, 17, 19, and 22 involve 2 or more hypoplastic arteries of the CW, in fact, 22.2% of USA2 [17] falls into these 4 configurations as compared to USA 1 [2]; 4.8%, Morocco [16]; 1% France [18]; 2%, Iran [19] 2.9% and 0.4% in the present study. In general, both USA studies [2,17] separate out from the non-USA studies [[16-18] and the present study] (Figure 3), with USA2 [17] separating out as above and USA1 [2] separating out from the rest due to differences in Configurations 2,8,9,14,15,16; for these configurations, many involving hypoplastic A1, USA1 [2] sums to around 20% while the others sum to 12.5% or less (see Figure 3 and Table 1). Note that these findings are in contrast to Eftekhar et al., 2006, [19] who studied CW of 102 male, deceased Iranian subjects and evaluated the distribution of configurations in the variations of the CW in different populations [2,16-18]; they did not find any racial variation.

Table 1 reveals that there is a marked variation in CW among ethnic and racial populations. There exist several postulates as to the underlying reasons for the anatomical variation of the CW among which are, selection of cases: brains obtained from those who have died of causes unrelated to the brain in [16,19] and the present study, and died of disease of the brain [2] and in unselected cases in [17]. Gender: male and female cases were studied in [16] and the present study, male only in [19]. Other studies [2,17,18] had not mentioned the sex distribution of their cases. The definition of hypoplasia was consistent in these studies [2,16-19] but the diameter of component vessels of the CW has not been performed in all the studies [2,18]. Prevalence of the "typical" configuration in the present study is 14.2% in 225 brains examined as compared to studies reported in India: 26.8% in Maharashtra, India[25] 53.2% in South India, [26] and 45.20% in Northwest India, [4] in 175, 357 and 1000 apparently normal brains examined respectively. The definition of hypoplasia was consistent in these studies [4,25,26] but the diameter of component vessels of the CW has not been performed in all the samples, and has not investigated the variations of CW as a whole. It is believed that Sri Lankans have a common origin from India, further studies are needed to ascertain reasons for the wide range in the prevalence of "typical" configuration between studies in India and Sri Lanka.

However, the methods and definition of hypopolasia differ among anatomical studies in the literature, which may hamper the comparison of these studies. Establishing an international standard method for nomenclature on the variation and for quantitative measurement of the diameters of all the component vessels of the CW and quantitatively define hypoplasia would make it possible for comparison of data with studies in diverse populations.

## Conclusion

The present study reveals that there are significant variations in the CW among intra and inter ethnic groups (Caucasian, African and Asian: Iran and Sri Lanka dominant populations), this is of clinical importance and warrants further studies to ascertain the influence of genetic, racial, regional, environmental and hemodynamic factors or a combination of any of the above keeping the methods of measurements, data assessment, and the definitions of hypoplasia the same.

## List of abbreviations

AcoA: Anterior communicating artery; ACA: Anterior cerebral artery; A1: pre communicating part of ACA; A2: post communicating part of ACA; CW: circle of Willis; ICA: Internal carotid artery; PCA: Posterior cerebral artery; P1: pre communicating part (P1) of PCA; P2: post communicating part (P2) of PCA; PcoA: Posterior communicating artery.

## Competing interests

The authors declare that they have no competing interests.

## Authors' contributions

KRDS carried out the data extraction, performed the analysis and drafted the manuscript. RS helped out with the data extraction WSLG and RWJ supervised the study and participated in its coordination and drafted the manuscript. DA did the statistical analysis and drafted the manuscript. All authors read and approved the final manuscript.

## Pre-publication history

The pre-publication history for this paper can be accessed here:

http://www.biomedcentral.com/1471-2377/11/5/prepub
